# Turkish reliability and validity study of the medical outcomes study (MOS) sleep scale in patients with obstructive sleep apnea

**DOI:** 10.3906/sag-1909-157

**Published:** 2021-02-26

**Authors:** Bülent Devrim AKÇAY, Duygu AKÇAY, Sinan YETKİN

**Affiliations:** 1 Department of Mental Health and Diseases, Sleep Research Center, Gülhane Education and Research Hospital, Ankara Turkey; 2 Department of Military Health Services, Ministry of National Defense, Ankara Turkey; 3 Department of Mental Health and Diseases, Sleep Research Center, University of Health Sciences, Ankara Turkey

**Keywords:** Medical outcomes study-sleep scale, obstructive sleep apnea, sleep quality, reliability, validity

## Abstract

**Background/aim:**

The purpose of the present study was to evaluate the Turkish reliability and validity of the medical outcomes study (MOS) sleep scale in patients who have obstructive sleep apnea.

**Materials and methods:**

The data of the study were collected from 120 adult patients with obstructive sleep apnea and from 90 healthy individuals between March 04 and May 31, 2019.

**Results:**

The Cronbach’s α internal consistency reliability coefficient of the MOS sleep scale was found as 0.82. The test-retest reliability was acceptable (r = 0.76-0.94). Six factors were identified by the factor analysis. These were the same as those in the original MOS-Sleep. The correlations between the MOS-Sleep and other instruments administered in this study provided evidence for structural validity. A significant relation was determined between MOS sleep scale and obstructive sleep apnea syndrome (OSAS) severity and the healthy group ( P < 0.05). In addition, the Cronbach α internal consistency reliability coefficient of the healthy group in MOS sleep scale was found as 0.78. The items of the six factors that were obtained with the confirmatory factor analysis for the MOS sleep scale of the healthy group were found to be the same as in the original MOS-Sleep.

**Conclusion:**

Turkish MOS sleep scale is a measurement tool that consists of 12 items and 6 subdimensions with adequate validity and reliability indicators.

## 1. Introduction 

Obstructive sleep apnea syndrome (OSAS), which is known to be the most common sleep disorder, is a clinical manifestation that is characterized with recurrent, partial, or complete obstruction of the upper respiratory tract during sleep. The most important symptoms of it are snoring, apnea, excessive drowsiness during the day, inadequate sleep, deterioration in attention, concentration, and memory [1,2]. OSAS is diagnosed with polysomnography (PSG), which is the gold standard diagnostic method. However, this method is expensive, laborious, and requires specially-equipped laboratory [3]. 

As an alternative, self-report methods like sleep questionnaires might be used to obtain data about sleep. These questionnaires are applied easily to ensure that sleep is evaluated in terms of quantity (latency) and quality (depth or restfulness of sleep). Several sleep questionnaires were developed in previous years to evaluate sleep disorder and quality [4-7]. In Turkey, the validity and reliability of the following studies were conducted previously: sleep hygiene index [8] to assess sleep hygiene [8], Pittsburg Sleep Quality Index to screen the sleep quality [9], the Epworth Sleepiness Scale to assess sleepiness during daily activities [10], and the STOP-Bang Test, which is often used by anesthesiologists for the investigation of OSAS in preoperative evaluations [11]. One of the most commonly used scales in evaluating broad-spectrum sleep quality is the Medical Outcomes Study-Sleep Scale (MOS-Sleep). The MOS sleep scale is not specific to the disease, consists of 12 items, and is a self-report scale to evaluate the data not only on sleep quality but also on sleep. The MOS sleep scale measures the subjective sleep experiences in six areas. Each area measures a different sleep dimension. It requires only 2 - 5 min to complete the scale [12].

Psychometric evaluation of the MOS Sleep Scale during development supported its use in the assessment of sleeping problems (and changes in sleeping problems) in both clinical and nonclinical populations [12]. The scale was also used commonly in various clinical populations like diabetic neuropathic pain, over-active bladder, postherpetic neuralgia, and restless leg syndrome [13,14,15]. In non-English speaking countries, various language versions of MOS sleep scale were evaluated, and the psychometric feature of it was reported to be good [16,17]. Korean versions of the MOS-Sleep have been recently evaluated in patients with OSAS [17]. 

In our country, a reliable and cost-efficient scale is needed to collect data on sleep conditions of patients with OSAS. For this reason, this study was planned to perform the reliability and validity of the Medical Outcomes Study (MOS) Sleep Scale Turkish Version in patients with obstructive sleep apnea.

## 2. Method

### 2.1. Subjects

The data were collected from 120 adult patients with OSAS (62.5% male; mean age: 47.6 years; range: 19 to 79 years), who referred to Ankara Gülhane Training and Research Hospital Sleep Research Center for the evaluation of suspected OSAS between 04 March 2019 and 31 May 2019. Ethical permission was received from Ankara Numune Training and Research Hospital Ethics Committee on May 08, 2018 with the decision number 1952/2018 before the study was commenced. It is recommended in scale validity and reliability studies that the number of items in the scale is 5-10 to determine the sample volume [18]. For this reason, the study was not terminated unless the number of the participants was 120. 

The main complaints of the patients were symptoms that were related to OSAS like snoring during night sleep, witnessed breathing stops, excessive daytime sleepiness, and inadequate sleep. The mother tongue of the patients that were included in the study was Turkish. The patients, who were over the age of 18, who were able to read and write, who agreed to participate in the study, and who underwent one night of polysomnography, were included in the present study. The patients who had active psychiatric, medical, or sleep disorders that would affect judgment or quality of life beyond the effects of OSAS were excluded from the study. The patients, who had depression, anxiety or psychosis, who were receiving regular medication like sleeping pills, antidepressants, anxiolytics or antipsychotics, were also excluded from the study. However, the patients who had subclinical depression or anxiety disorders and the patients with hypertension or diabetes without excessive cardiovascular complications were not excluded. Table 1 shows the demographic characteristics in detail. Among the OSAS patients included in the study, 19 (15.8%) patients had diabetes and 22 (18.3%) patients had hypertension. Successive ≥4 movements that lasted 5-90 s were accepted as periodic leg movement (18). The patients who had five or more stimuli per hour that were associated with periodic leg movements during sleep were not included in the study.

**Table 1:** Characteristics of the subjects included.

Gender	nN	%
Male	75	62.5
Female	45	37.5
Age (Mean±SD)	47.56±13.32	
Educational status	n	%
Primary school	31	25.8
Secondary school	35	29.2
University and above	54	45.0
Working status	n	%
Yes	67	55.8
No	53	44.2
Marital status	n	%
Married	94	78.3
Single	26	21.7
Income status		
Low	37	30.8
Moderate	74	61.7
High	9	7.5
Apnea-hypopnea index (AHI)	n	%
5/h ≤AHI < 15 /h	38	31.7
15/h ≤ AHI < 30 /h	49	40.8
AHI ≥ 30 /h	33	27.5
Polysomnographic parameters (Mean±SD)		
N1 (min)	99.07±58.32	
N2 (min)	143.75±54.17	
N3 (min)	41.59±26.71	
R (min)	44.22±24.28	
TST (min)	331.97±70.91	
WASO (min)	60.15±55.51	
Sleep efficiency (%)	79.75±16.02	
Average O2 Saturation	85.42±74.73	

TST: total sleep time; WASO: wake after sleep onset.

### 2.2. Data collection tools

#### 2.2.1. MOS sleep scale

The MOS sleep scale, which consists of 12 items, measures the subjective sleep experiences of individuals in several different areas. The scale is a nondisease-specific tool used in evaluating sleep results based on the self-reports of the patient. In practice, participants are asked to remember the last 4 weeks, and answer the related questions. Ten out of 12 questions require 6-point Likert-type answers and 1 requires 5-point Likert-type answer, and the participants are asked to write the average sleeping hour in the question of the amount of sleep.

The scale was designed to include the items, which would measure some sleep characteristics defined among different sleep-related diseases or syndromes. In the MOS sleep scale, the Sleep Problems Index and six subdimension scores are given. The domains are: (1) sleep disturbance, which comprised 4 items (Q1, Q3, Q7, and Q8); (2) sleep adequacy, which comprised 2 items (Q4 and Q12); (3) sleep quantity, which comprised 1 item (Q2); (4) somnolence, which comprised 3 items (Q6, Q9, Q11); (5) snoring which comprised 1 item (Q10); and (6) shortness of breath, or headache, which comprised 1 item (Q5). In addition, the mean score of questions 1, 3, 4, 5, 6, 7, 8, 9, 12 and Sleep Problem Index are also calculated in this respect. The scale is scored by converting the scores of the Sleep Problem Index scores and subdimensions into a scale of 0 to 100. If the participant specifies the “sleep amount” subdimension as 7 or 8 sleeping hours, “1” point is given, and the other durations are scored as “0”. High scores in the sleep disorder, somnolence areas and Sleep Problems Index scores indicate that the sleep problem of the patient is more severe. However, low scores in the sleep amount and sleep adequacy show more serious sleep problems [12]. 

#### 2.2.2. Sleep hygiene index (SHI): 

Its Turkish validity and reliability study was conducted by Özdemir et al. [8]. It consists of 13 questions and is a 5-point Likert scale. This index aims to evaluate the presence of sleep hygiene through questioning how often the patient has sleep behaviors constituting sleep hygiene. The scores range between 13 and 65; and higher scores indicate poorer sleep hygiene for the participant.

#### 2.2.3. Pittsburg sleep quality index (PSQI):

PSQI is a self-report scale that was adapted into Turkish by Agargün et al. [9] and consists of 19 items evaluating the sleep quality and sleep disorder in the past 1 month. It has 24 questions, 19 of which are in the form of self-repot, and 5 of which are answered by the spouse or roommate of the patient. The scale consists of 7 components, which are subjective sleep quality, sleep latency, sleep duration, usual sleep activity, sleep disorder, use of sleeping pills, and daytime dysfunction. Each component is evaluated over a score of 0-3. The total score of these seven components yields the total score of the scale, and the total score ranges between 0 and 21. The total score being bigger than 5 shows “poor sleep quality”.

#### 2.2.4. Epworth sleepiness scale (ESS):

The ESS whose validity and reliability were carried out by Ağargün et al. [10] is a 4-item Likert-style scale. It is scored as 0, 1, 2 and 3, and 10 points and above show excessive daytime sleepiness. The probable total score varies between 0 and 24. High scores show that there is more sleepiness during daily activities [10]. 

#### 2.2.5. Beck depression inventory (BDI): 

The Turkish validity and reliability study of this scale was conducted by Hisli in 1989, and its cut-off point was defined as 17. The scale consists of 21 items, and each item is scored over 0-3 points. The total score varies between 0 and 63. High scores represent high depression levels [19].

#### 2.2.6. Beck anxiety inventory (BAI): 

The validity and reliability study of the scale was conducted by Ulusoy et al. [20] in Turkey. It is a self-evaluation scale used to determine the frequency of the anxiety symptoms of an individual. The scale consists of 21 items and is scored between 0 and 3 in Likert-style. High scores represent high anxiety levels. 

#### 2.2.7. Short form-36 health survey (SF-36) 

SF-36 was developed to evaluate quality of life, and consists of 36 questions on physical function, the role of the limitations caused by physical health problems, the role of the limitations caused by mental problems, energy/exhaustion, mental well-being, social functions, pain, and general health. All fields of the scale are converted into scores ranging from 0 (the lowest function level) to 100 (the highest function level) in the evaluation. Higher score indicates a better quality of life related to health [21]. The Turkish version of the SF-36 was validated.

#### 2.2.8. Checklist individual strength survey (CIS survey): 

According to this scale, fatigue is evaluated according to four aspects, which are: subjective fatigue perception, decrease in concentration, decrease in motivation, and decrease in physical activity. The questionnaire consists of 20 statements that measure the fatigue in the past 2 weeks, and a 7-point Likert-type scale is used for answers. This scale evaluates fatigue in four aspects, which are: subjective experience, reduced motivation, reduced activity, and decreased concentration. Higher scores show that the fatigue has increased [22]. The Turkish version of CIS was also validated.

#### 2.2.9. Polysomnography

Polysomnography was performed with the Grass Comet Plus AS40 device. Electroencephalography (EEG, F3-M2, C3-M2, O1-M2, F4-M1, C4-M1, O2-M1), electrooculography (EOG), submental electromyography (EMG), bilateral anterior tibialis electromyography, and electrocardiography (ECG) recordings were performed in accordance with the American Academy of Sleep Medicine (AASM) criteria. Respiratory inductive plethysmography belts recorded the chest and abdominal movements. Airway flow was evaluated with a nasal airway and thermistor. Pulse rate and oxygen saturation were measured by a finger probe oximeter. Polysomnography was applied to all cases in the same protocol. The records were scored by an experienced sleep physician according to the standard AASM criteria, without knowing whether patients had OSAS [23]. 

### 2.3. Process

**Figure 1:**
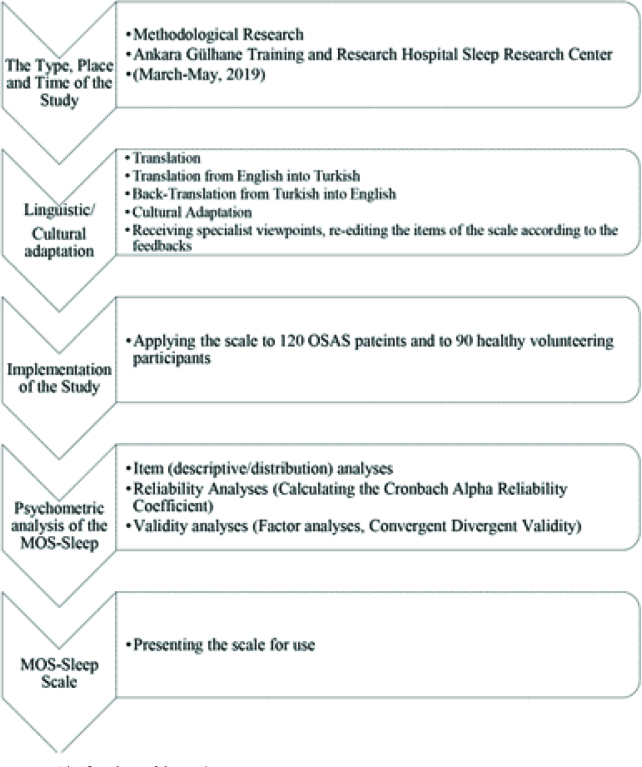
The flowchart of the study.

The flowchart of the study is given in Figure 1.

#### 2.3.1. Linguistic/cultural adaptation

*Translation*

Permission was obtained from Prof. Ron D. Hays for the Turkish adaptation of the MOS sleep scale. The original name of the scale, which is in English, was translated into Turkish by the researchers and two people who were specialists in language field separately. Then, the researchers prepared a Turkish text by analyzing the most suitable translation of each item. After the required editing was made, the scale was translated into English with the back-translation method. After this translation, the sentences in the original text and in the retranslation were compared by the researchers, and the statements that were not understandable were reedited and the form was made ready for specialist viewpoints. 

*Cultural adaptation*

For the purpose of evaluating the validity of the scale, the content validity was made by applying expert opinion method after the language adaptation was carried out. In this respect, the scale was presented to receive the viewpoints of three experts who had studies released in the literature. The experts were asked to evaluate the scale in terms of the suitability of the translated form of the expressions to the original form, appropriateness of the expressions in terms of understandability to the target group, its adequacy to evaluate sleep disorder of OSAS patients; they considered it necessary and expressed their opinions by giving explanations. The opinions, suggestions, and criticisms of the experts about the items in the scale obtained from the evaluation forms were evaluated and the articles were reedited. The scale that was applied is given in Appendix 1.

#### 2.3.2. Psychometric analysis of the MOS-Sleep

##### 2.3.2.1. Item (descriptive/distribution) analyses

Firstly, the central tendency, dispersion, and distributions of all MOS sleep scale items were investigated. 

##### 2.3.2.2. Reliability analyses

Internal consistency and test-retest reliability of the MOS sleep scale were evaluated to test the reliability. The internal consistency of the scale was tested with Cronbach’s α. It is recommended that α value is at least 0.70 to be considered reliable [24]. A two-week period was chosen between each assessment to minimize the recall of the subject’s previous responses to examine the test-retest reliability. The first data of the MOS sleep scale were obtained when participants visited the sleep laboratory for a night sleep, and retesting was performed before the intervention procedures (i.e. positive air pressure titration or sleep-related drugs) were administered. The scale was applied to 90 participants twice at two-week intervals for test-retest reliability.

For the purpose of examining how well the elements of each area represent a particular characteristic compared to other characteristics, the item convergence and discrimination of the MOS sleep scale was also assessed. The item convergence evaluates the correlation between each item, and its being bigger than 0.40 is considered to be adequate to meet the criterion [25]. The item discrimination requires that the domain elements have higher correlations with the elements in their own domains than those in other domains [26]. The strong correlation percentage with items that are outside its own domain leads to the questioning of the assumptions of the scale. 

##### 2.3.2.3. Validity analyses

###### 2.3.2.3.1. Criterion validity 

*The relation of MOS sleep scale with the healthy group*

The data of the study were collected from 90 healthy individuals (67.8% male; mean age: 44.5 years; age range: 19-73 years; educational status: 43.8% university and above; 58.5% working; 80.2% married; income status: 62% moderate;), who referred to Ankara Gülhane Training and Research Hospital as patient relatives and who had no history of neurological, psychiatric, or sleep disorder diagnoses and treatment between 04 March 2019 and 31 May, 2019. In the healthy group, OSAS symptoms and findings were investigated by using nonpolysomnography diagnosis methods (physical examination and anamnesis). The independent sample t-test was employed to determine the differences between the MOS sleep scale subgroup mean scores of OSAS and the healthy group.

*The relation of MOS sleep scale with the severity of OSAS*

The Apnea-Hypopnea Index (AHI), which is an objective assessment, was selected to evaluate the relation between the MOS sleep scale and the severity of OSAS. A one-way analysis of variance was employed for categorical analysis of AHI with the MOS sleep scale.

###### 2.3.2.3.2. Construct validity 

####### 2.3.2.3.2.1. Factor analyses 

*Confirmatory factor analysis*

In the present study, the confirmatory factor analysis was applied. The AMOS 23 (IBM Corp., Armonk, NY, USA) program was used for confirmatory factor analysis.

####### 2.3.2.3.2.2. Convergent-divergent validity

To test the convergent validity, the scale is applied simultaneously with another scale examining the same or associated structure that is previously proven to be valid. In this study, MOS sleep scale and PSQI, CIS, BDI, BAI, ESS, SHI, and SF-36 scales were applied simultaneously. The Pearson correlation analysis was made to identify the relations between them.

## 3. Results

### 3.1. Item (descriptive/distribution) of the MOS-Sleep

All items were examined in terms of the means and distributions according to the “-/+ 2” rule [27] (Table 2). Skewness and Kurtosis scores were evaluated as normal distribution. 

**Table 2:** The descriptive statistics of Turkish version of MOS-sleep items.

	Min	Max	M	SD	Skewness	Kurtosis
MOS Sleep 1	1	5	3.88	1.22	-1.023	0.169
MOS sleep 2	2	10	6.37	1.54	-0.039	0.949
MOS sleep 3	1	6	3.74	1.50	-0.212	-1.193
MOS sleep 4	1	6	2.92	1.45	0.452	-0.793
MOS sleep 5	1	6	4.03	1.53	-0.187	-1.126
MOS sleep 6	1	6	3.55	1.56	0.041	-1.214
MOS sleep 7	1	6	3.95	1.73	-0.408	-1.129
MOS sleep 8	1	6	3.86	1.57	-0.307	-1.040
MOS sleep 9	1	6	3.98	1.58	-0.257	-1.056
MOS sleep 10	1	6	2.29	1.47	1.033	0.020
MOS sleep 11	1	6	3.76	1.66	-0.257	-1.078
MOS sleep 12	1	6	3.29	1.63	0.214	-1.190

### 3.2. Reliability of the MOS-Sleep

The Cronbach’s α internal consistency reliability coefficient of the MOS sleep scale was found to be 0.82. The internal consistency reliability coefficients of the subdimensions of the scale were found to be between 0.79 (daytime somnolence) and 0.91 (sleep disturbance). It was also determined that the subdimensions of the MOS sleep scale and Sleep Problems Index internal consistency levels were good. The test-retest correlation coefficients of the scale varied between 0.76 (sleep quantity) and 0.94 (daytime somnolence), and the scale was considered to be reliable (Table 3).

**Table 3:** Internal consistency and test-retest reliability of Turkish version of MOS-sleep.

Domain	MOS-Sleep items	Scale mean if item deleted	Scale variance if item deleted	Corrected Item-Total Correlation	Cronbach’s Alpha if item deleted	Cronbach’s Alpha(n = 120)	Test-retest r(n = 90)	Mean explained variance	Mean(SD)
Sleepdisturbance	MOS-Sleep 1	11.55	19.93	0.66	0.92	0.91	.89**	28.58	33.22 (22.12)
	MOS-Sleep 3	11.69	16.90	0.76	0.89				
	MOS-Sleep 7	11.48	14.02	0.89	0.84				
	MOS-Sleep 8	11.58	15.51	0.86	0.85				
Snoring	MOS-Sleep 10	NA					.80**		74.17 (29.37)
Shortness ofbreath	MOS-Sleep 5	NA					.84**		39.50 (30.51)
Sleepadequacy	MOS-Sleep 4	3.29	2.66	0.70	NA	0.82	.87**	8.08	42.08 (28.43)
	MOS-Sleep 12	2.92	2.09	0.70	NA				
Daytimesomnolence	MOS-Sleep 6	7.73	7.69	0.70	0.63	0.79	.94**	16.15	44.78 (26.79)
	MOS-Sleep 9	7.31	8.23	0.60	0.74				
	MOS-Sleep 11	7.53	7.92	0.59	0.75				
Sleep quantity	MOS-Sleep 2	NA					.76**		6.37(1.54)
Sleep problemsindex	MOS-Sleep 1	29.31	74.60	0.50	0.84	0.85	.92**	86.59	44.64 (21.09)
	MOS-Sleep 3	29.45	66.87	0.71	0.82				
	MOS-Sleep 4	30.28	70.67	0.57	0.83				
	MOS-Sleep 5	29.17	75.67	0.42	0.86				
	MOS-Sleep 6	29.64	71.43	0.48	0.84				
	MOS-Sleep 7	29.24	63.98	0.71	0.82				
	MOS-Sleep 8	29.33	66.29	0.70	0.82				
	MOS-Sleep 9	29.22	69.31	0.56	0.83				
	MOS-Sleep 12	29.90	68.53	0.57	0.83				

NA: not applicable, MOS-Sleep: medical outcomes study-sleep scale, SD: standard deviation.

The items of the domain correlations were calculated for 9 items comprising three domains such as sleep disturbance (0.66-0.86), sleep adequacy (0.70-0.70), and daytime somnolence (0.59-0.70). Correlations of the items with sleep problems indices ranged from 0.42 to 0.71. With regard to item discrimination, all items had a higher correla­tion with their own domains than they did with others.

### 3.3. Validity of the MOS-Sleep

#### 3.3.1. Criterion validity 

*The relation of MOS sleep scale with the healthy group*

A significant relation was detected between the shortness of breath, sleep disturbance, snoring and sleep adequacy subdimensions of the MOS sleep scale of the OSAS group and the healthy group ( P <0.05). The Cronbach’s α internal consistency reliability coefficient of the MOS sleep scale of the healthy group was determined as 0.78. The internal consistency reliability coefficients of Cronbach’s α of the subdimensions were determined as daytime somnolence: 0.73; sleep quantity: 0.59; sleep disturbance: 0,79; sleep problems index: 0.79. It was determined that the healthy group had adequate sampling size for factor analysis (the KMO value: 0.73; Bartlett test result: ×2 = 242.338; P < 0.001). It was determined that the items of the six factors [28,29] —together with single-item dimensions excluded from the analysis— were the same with the original MOS sleep scale.

*Relationship of MOS-Sleep to the severity of OSAS*

The patients were divided into 3 groups according to the severity of AHI as the mild group (5/h ≤AHI<15/h), moderate group (15/h ≤AHI<30/h), and severe group (AHI≥30/h). A significant relation was detected between the snoring subdimension scores of the MOS sleep scale (P < 0.05) and the severity of AHI. It was determined that this difference was between the mild and severe groups in the analysis that was made to understand the difference between the groups.

#### 3.3.2. Construct validity 

##### 3.3.2.1. Factor analyses 

*Confirmatory factor analysis*

Confirmatory factor analysis was applied to examine the factor structure of the scale. The Kaiser-Meyer-Olkin (KMO) value was calculated as 0.80, and the Bartlett test result was calculated as x2 = 637.035, P = 0.01 in the present study. According to the criteria that were determined, it was found that the sampling size was adequate for factor analysis in this study. In line with the literature [28,29], the items of the three single-item subdimensions in the scale (sleep quantity: item number on scale is 2; snoring: item number on scale 10; and shortness of breath, or headache: item number on scale is 5) were not included in the factor analysis. 

The Chi-square value was calculated as 48.035 (P < 0.1), and the rate of it to the degree of freedom (24) was found to be 48.035/24 = 2.0. The fact that this value is 5 shows a good fit [30]. The resulting value shows that the goodness of fit of the measuring model is at a good level. 

**Figure 2:**
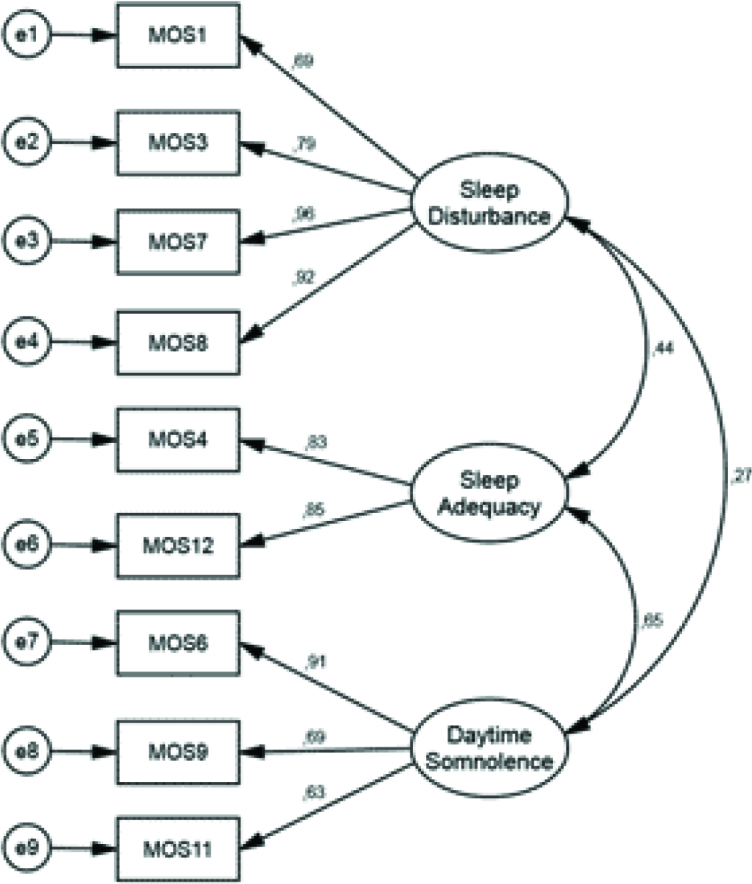
Graphical representation of the confirmatory factor analysis of the MOS sleep scale.

The root mean square error of approximation (RMSEA), which was obtained as a result of the analysis, was found to be .09. Tabachnick and Fidel [31] reported that the RMSEA value being <0.10 is an acceptable level of goodness of fit. The goodness of fit index (GFI), which was calculated after the analysis, was .92, and the adjusted goodness of fit index (AGFI) value was .85. According to the literature, the >0.90 is acceptable GFI criterion for GFI, and >0.80 is acceptable for AGFI [32,33]. Three subdimensions were obtained from these included items. These factors were: (1) sleep disturbance, (item numbers on scale are 1, 3, 7, 8); (2) sleep adequacy, (item numbers on scale are 4, 12); (3) somnolence, (item numbers on scale are 6, 9, 11) . As a result, 12 items and 6 subdimensions were obtained in the Turkish MOS sleep scale, as in the original MOS sleep scale. Figure 2 is a graphic representation of confirmatory factor analysis.

##### 3.3.2.2. Convergent divergent validity

Table 4 presents the correlation coefficients of the MOS sleep scale with the other instruments applied in this study. A moderate and positive relation was detected between the sleep disturbance and PSQI; daytime somnolence and CIS and ESS; sleep problems index and CIS, BDI, and BAI (0.50-0.69). A moderate and negative relation was detected between the energy/viability/vitality subdimension and daytime somnolence and sleep problems index of the SF 36 Scale. A strong (0.70-0.89) and positive relation was detected between the sleep problems index and PSQI. It was determined that there were poor or no relations between the sleep quantity and snoring subdimensions and the other scales that were used. 

**Table 4:** Correlation coefficients between MOS-Sleep and other questionnaires.

Domain	PSQI	CIS	BDI	BAI	ESS	SHI	SF36							
							Physical function	Physical role difficulty	Pain	General health perception	Energy/viability/vitality	Social functionality	Emotional role difficulty	Psychological health
Sleep disturbance	.635**	.389**	.460**	.393**	-0.068	.230*	-0.059	-.344**	-.309**	-.225*	-.419**	-.354**	-.380**	-.379**
Snoring	0.088	.238**	0.162	.229*	.327**	.219*	-0.063	0.027	-0.042	-0.076	-.197*	-0.027	0.066	-0.174
Shortness of breath	.194*	.259**	.380**	.343**	.207*	.189*	0.042	-0.073	-.232*	-.262**	-.280**	-.227*	-0.078	-.200*
Sleepadequacy	-.470**	-.435**	-.458**	-.353**	-.239**	-.247**	0.026	.269**	.260**	.237**	.510**	.363**	.228*	.374**
Daytime somnolence	.437**	.539**	.427**	.424**	.509**	.388**	-.407**	-.347**	-.353**	-.416**	-.513**	-.436**	-.351**	-.351**
SleepQuantity	-.382**	-0.094	-0.101	-0.037	-0.037	-0.107	0.096	0.165	.197*	0.113	0.085	0.154	0.097	0.033
Sleep Problems Index	.697**	.548**	.589**	.524**	.195*	.364**	-0.144	-.417**	-.409**	-.388**	-.601**	-.482**	-.429**	-.479**

* P < 0.05. **P < 0.01.

**Table 5:** MOS UYKU ÖLÇEĞİ

Son dört hafta boyunca ne kadar sıklıkla …		(Her bir satırdan bir tek rakamı yuvarlak içine alarak işaretleyin)					
		Her zaman	Çoğu zaman	Biraz	Bazen	Çok az	Hiçbir zaman
3.	uykunuzun rahat olmadığını hissettiniz? (uyurken huzursuz bir şekilde hareket etmek, gergin hissetmek, konuşmak, vs.)	1	2	3	4	5	6
4.	sabah uyandığınızda kendinizi dinlenmiş hissedecek kadar uykunuzu aldınız?	1	2	3	4	5	6
5.	nefes darlığı veya baş ağrısıyla uyandınız?	1	2	3	4	5	6
6.	kendinizi gün boyunca uykulu/uyku sersemi ya da uyuşuk hissetiniz?	1	2	3	4	5	6
7.	uykuya dalmakta zorluk çektiniz?	1	2	3	4	5	6
8.	gece uyandığınızda yeniden uykuya dalmakta güçlük çektiniz?	1	2	3	4	5	6
9.	gün boyunca uyanık kalmakta sorun yaşadınız?	1	2	3	4	5	6
10.	uykuda horladınız?	1	2	3	4	5	6
11.	gün içerisinde şekerleme yaptınız?(5 dakika veya daha uzun)	1	2	3	4	5	6
12.	ihtiyacınız olan uykuyu aldınız?	1	2	3	4	5	6

## 4. Discussion

In the validity and reliability studies that were conducted in previous years, it was reported that the internal consistency reliability of the scale did not reach the threshold in some countries in sleep quantity and daytime somnolence subdimensions [12,16,17]. It was determined in this study that the internal consistency reliability of the Turkish version of the Cronbach’s MOS sleep scale was excellent in all subdimensions. It was also determined that the scale has a high reliability.

In the validity and reliability study of Kim et al. [17] in Korean language, the findings on the test-retest reliability evaluations were determined as 0.47-0.87. In this study, the test-retest correlation coefficients of the scale were found to be better (0.76-0.94). For this reason, the Turkish version of the MOS sleep scale is considered to have acceptable test-retest reliability.

Published factor analysis data for nonEnglish versions of the MOS sleep scale exist only in one study. As it was the case in the validity and reliability study conducted with OSAS patients in Korean language [17], it was determined in our study that the items of the six factors were the same as the original MOS sleep scale, together with single-item dimensions that were excluded from the analysis. Meanwhile, after excluding three subdimensions that consisted of single item, it was determined in the confirmatory factor analysis that the goodness of fit value of the measuring model was good. For this reason, it was determined that the Turkish version of the MOS sleep scale measures all of the cohesive factors that exist in the original version of the MOS sleep scale.

In the present study, it was determined that all of the items that were in the scale subdimension and sleep problems index exhibited item-scale correlation that was higher than 0.40 for the hypothesized dimension [25]. In other words, it was determined that the correlation levels between each item and their domains were good. Also, the item discrimination of the scale was satisfactory. The scaling success rates on discriminant validity in the sleep disturbance, sleep adequacy and daytime somnolence subdimensions of the scale were 100%. All of the items showed lower item correlations (less than 0.40) with other domains, which shows that the Turkish MOS sleep scale items are more strongly related to their hypothetical dimensions than the other dimensions of the scale. In previous studies that were conducted in different languages, the item convergence validity results were evaluated as satisfactory [16,17].

In previous years, the relations between OSAS and neuropsychological and functional deficiencies including daytime sleepiness, impaired sleep quality, fatigue, anxiety, depression and reduced quality of life have been shown [34,35]. For this reason, the MOS sleep scale and PSQI, CIS, BDI, BAI, ESS, SHI and SF-36 scales were applied simultaneously.

It was determined that there is a relation between the 9-item Sleep Problems Index of the MOS sleep scale and the scales that were applied in the study except for the physical function subdimension of the SF-36. It was also determined that this relation was negative with SF-36 subdimensions, and positive with other scales. These findings are consistent with the results of the previous studies reporting that sleep problems of the OSAS patients affect mental and social functioning as well as physical health in a negative way [35-39]. It was considered that there is an agreement between the MOS sleep scale result and the scales that were applied simultaneously. 

The comparison results of the subdimensions of the MOS sleep scale AHI (snoring) and healthy group (shortness of breath, sleep disturbance, snoring and sleep adequacy) are effective in distinguishing the patients who have OSAS. In addition, the Cronbach’s α values and the factor analysis results of the healthy group show that the MOS sleep scale is an adequate scale for the measurement and evaluation of adults in general population.

The inclusion of the patients with OSAS who underwent polysomnography, which is the gold standard diagnostic method, is considered as one of the superior aspects of the present study. Using more than one scale for simultaneous validity comes to the forefront as another superiority of the study. It is considered as a limitation of the study that OSAS symptoms and findings were found not to exist in the healthy group by using nonpolysomnography diagnostic methods (physical examination and anamnesis). We believe that it would be appropriate to evaluate the present study by considering this limitation. 

As a conclusion, the adaptation study of the Turkish version of the MOS sleep scale on Turkish patients with OSAS-OSAS showed that the scale is valid and reliable at an adequate level. In addition, the Turkish version of the MOS sleep scale was also found to be a beneficial scale in assessing important aspects of the perceived sleep in adults in general population. It was determined that the MOS sleep scale is a practical, easy-to-apply, and assessable scale, which can be used in this field.

## Informed Consent

Informed consent was obtained from all individual participants included in the study.
